# Characterization of TMEM16F-Specific Affibodies and Their Cellular Effects

**DOI:** 10.3390/membranes15090255

**Published:** 2025-08-28

**Authors:** Eunyoung Kim, Jinho Bang, Sunghyun Kim, Byoung-Cheol Lee

**Affiliations:** 1 Neurovascular Unit Research Group, Korea Brain Research Institute, Daegu 41068, Republic of Korea; eunyoun9020@kbri.re.kr; 2 Bio-Healthcare Materials Center, Korea Institute of Ceramic Engineering and Technology, Cheongju 28160, Republic of Korea; muhonmania@naver.com (J.B.); shkim0519@kicet.re.kr (S.K.)

**Keywords:** TMEM16, scramblase, channel, affibody, biopanning

## Abstract

The TMEM16 (Anoctamin) family comprises a group of transmembrane proteins involved in diverse physiological processes, including ion transport and phospholipid scrambling. TMEM16F (Anoctamin 6), a phospholipid scramblase and nonselective ion channel, plays a central role in membrane remodeling, blood coagulation, immune responses, and cell death pathways through its ability to externalize phosphatidylserine in response to elevated intracellular calcium levels. Consequently, modulating TMEM16F activity has emerged as a promising strategy for the development of new therapeutic applications. Despite the functional importance of TMEM16F, TMEM16F modulators have received little study. In a previous study, we generated TMEM16F-specific affibodies by biopanning a phage display library for affibodies that bind to brain-specific TMEM16F (hTMEM16F) variant 1. In this study, we selected six other affibodies from among the 38 previously sequenced affibody candidates and characterized them. After purification, we confirmed that two of these affibodies bound to human TMEM16F with high affinity. To provide functional insights into how these affibodies modulate TMEM16F activity, we tested whether they could exert functional effects at the cellular level. Finally, we show that TMEM16F affibody attenuated the neuronal cell death induced by glutamate and microglial phagocytosis, suggesting that these affibodies might have potential therapeutic and diagnostic applications.

## 1. Introduction

The plasma membrane (PM) is a dynamic and highly organized structure that plays a fundamental role in maintaining cellular homeostasis and enabling communication with the extracellular environment. A hallmark feature of the PM is the asymmetric distribution of lipids between its inner and outer leaflets [1]. For instance, phosphatidylserine and phosphatidylethanolamine are mainly enriched in the inner leaflet, while phosphatidylcholine and sphingomyelin are typically found in the outer leaflet. This lipid asymmetry is tightly regulated by flippases and floppases, which actively maintain the differential distribution of specific lipid species across the bilayer [1,2]. In contrast to these ATP-consuming processes, lipid scrambling refers to a rapid, bidirectional movement of lipids between the membrane leaflets, which disrupts the asymmetric distribution of the lipids. It is mediated by a distinct class of proteins known as lipid scramblases [1]. The redistribution of lipids via scramblases serves as a key cellular signal in various biological contexts. The most well-characterized example is the exposure of phosphotidylserine on the outer leaflet of apoptotic cells, which serves as an “eat-me” signal for phagocytes, including macrophages and microglia [2,3].

According to recent reports, lipid scramblases can be broadly classified into three-types based on their activation mechanisms: those activated by caspases, those activated by calcium ions, and those that exhibit constitutive activity independent of external stimuli [4,5,6,7]. Among the identified scramblases, members of the TMEM16 family have emerged as calcium-activated lipid scramblases. TMEM16F, also known as anoctamin 6, is a calcium-activated phospholipid scramblase and ion channel that plays a crucial role in various physiological processes including blood coagulation, immune cell signaling, and membrane repair [8,9,10]. The proper regulation of TMEM16F activity is essential for maintaining cellular homeostasis, and thus dysregulation of TMEM16F has been implicated in Scott syndrome, inflammatory disorders, neurodegenerative diseases, and viral infections such as SARS-CoV-2 [11,12,13,14].

Given the physiological significance of TMEM16F, there is increasing interest in identifying and characterizing small molecules or biologics that modulate its activity. Such modulators could provide valuable tools for manipulating TMEM16F function and lead to the development of novel therapeutics. So far, however, studies of TMEM16F modulators have been limited, and even those reported have revealed specificity issues due largely to structural similarities between TMEM16 family members. This poses challenges for their uses as targeted tools and therapeutics. For instance, 1PBC is a potent TMEM16F inhibitor, but it also inhibits TMEM16A [15,16]. Anthelmintic niclosamide has also been reported to inhibit both TMEM16A and TMEM16F [14,17,18,19]. Moreover, recent studies have reported that niclosamide, a previously reported TMEM16A inhibitor, actually potentiates TMEM16A activity under physiological conditions [20]. Another TMEM16F inhibitor, A6-001, could still inhibit TMEM16A activity at high concentration [21]. For these reasons, further research is required to develop more specific modulators of TMEM16F proteins. Fortunately, due to the advances in cryo-electron microscopy, the binding sites of compounds to TMEM16F and/or TMEM16A are now known, which provides new opportunities for the rational design of selective modulators [15,16]. Continued research into TMEM16F modulators not only holds promise for the development of novel therapeutic strategies targeting TMEM16F-mediated pathways but also deepens our understanding of the complex role of TMEM16F proteins in cellular physiology.

In this study, we investigated whether TMEM16F-specific affibodies could serve not only as binding probes but also as functional modulators of TMEM16F proteins in cellular level. To do this, we selected six other TMEM16F affibodies and characterized their specificity and functional effects on TMEM16F proteins. Using various functional assays, we demonstrated that TMEM16F-specific affibodies are effective in modulating TMEM16F activity at the cellular level. These findings may not only provide valuable tools for basic research but also may lay the groundwork for therapeutic applications of TMEM16F in relevant diseases.

## 2. Materials and Methods

### 2.1. Purification of Human TMEM16 Protein

Human TMEM16F protein (Accession number, NM_001025356.2), human TMEM16A (XM_011545127), and TMEM16K (NM_018075) were tagged with SBP at their C-termini to facilitate their purification. After transfection into GnTI−cells (ATCC) using polyethylenimine (PEI) for 48–72 h, cells were harvested and resuspended in lysis buffer (150 mM NaCl, 20 mM HEPES, pH 7.4). The cells were then homogenized with a Dounce homogenizer and sonicated for 1 min, and 1% digitonin was added to facilitate extraction of human TMEM16. The extract was passed through a Streptavidin Plus Ultralink column in the presence of 0.06% GDN instead of 1% digitonin, and bound TMEM16F protein was eluted with 5 mM biotin. TMEM16 protein was further purified by FPLC on a Superose 6 column.

### 2.2. Purification of TMEM16F Affibodies

The DNAs of six candidate affibodies were synthesized and then inserted into the bacterial expression vector, pBT7-C-His (Bioneer, Daejeon, Republic of Korea). Plasmid DNAs were transformed into BL21(DE3). The cells were grown to an OD of ~0.6–0.8 and protein expression was induced by the addition of 1 mM IPTG for 3 h. Then, cells were resuspended in lysis buffer (150 mM NaCl, 20 mM Tris-Cl, pH 8.0). Proteins were purified using Talon resin (Takara). To reduce non-specific binding, resins were washed with 1 M NaCl and 20 mM imidazole and the affibody was eluted with 100 mM imidazole. After removing the remaining imidazole using a PD-10 desalting column (Cytiva, Marlborough, MA, USA), affibody proteins were concentrated using centrifugal filters (Thermo, Waltham, MA, USA).

### 2.3. Cell Cytotoxicity Assays and Cell Imaging

After seeding the HT-22 cells (MERCK) into poly-L-lysine coated 96-well plates (1 × 10^4^/each well), cells were incubated overnight. Then, 10 mM glutamate (pH 7.4) in the presence or absence of the affibodies was added to each well and incubated for 14 h at 37 °C to induce ROS-mediated cell damage. Before measuring LDH release, bright field images were obtained using an inverted Nikon ECLIPSE Ts2 microscope. Cell cytotoxicity was measured using the EZ-LDH assay kit (DoGenBio, Seoul, Republic of Korea), according to the manufacturer’s introduction. After centrifugation at 600× *g* for 5 min, the supernatant was collected to measure LDH release. Absorbance at 450 nm was used to measure cell cytotoxicity.

### 2.4. Biolayer Interferometry (BLI)

For the measurement of affibody binding to TMEM16 proteins, Octet N1 (Sartorius, Göttingen, Germany) was used as described in previous study [22]. The binding assay using BLI instrument was performed at 25 °C. Before starting the experiment, a streptavidin (SA) Biosensor was hydrated with deionized water for 10 min. The initial baseline was acquired by incubating the biosensor with buffer containing 150 mM NaCl and 20 mM HEPES, pH 7.4, for 30 s. Then 4 mL of TMEM16 proteins (3 mM) tagged with SBP was loaded onto the Octet Streptavidin (SA) biosensors for 150 s. After loading, the baseline was re-acquired for 150 s. To measure the association of the affibody with TMEM16 protein, 4 mL of affibody was loaded for 150 s. Finally, the dissociation of the affibody was monitored by transferring the biosensor back into buffer containing 150 mM NaCl and 20 mM Tris-Cl, pH 8.0. To calculate the Kd value for each affibody, the data were analyzed with BLItz Pro 1.3 software (ForteBIO, Fremont, CA, USA) using global fitting (1:1) according to the manufacturer’s instructions.

### 2.5. Phagocytosis Assay and Imaging

A phagocytosis assay was conducted using the Vybrant^TM^ phagocytosis assay kit (Thermo, Waltham, MA, USA) according to the manufacturer’s protocol. A 96-well plate was coated with poly-L-lysine to increase cell attachment. After seeding the plates with BV2 cells (1 × 10^5^/each well), purified affibodies were added to the cells for 1 h at 37 °C. After removing the medium, the cells were incubated for 2 h at 37 °C with fluorescein-labeled *E. coli* bioparticle^TM^. Finally, the remaining bacterial particles were aspirated, and Trypan Blue was added to each well to quench the fluorescence of the non-internalized bioparticles. The fluorescence measurements were performed using Flexstation3 (Molecular Device, San Jose, CA, USA) at an excitation wavelength of 488 nm and an emission wavelength of 520 nm. Four replicates were conducted to minimize experimental errors. Fluorescent images were acquired from the same 96-well plate after measuring fluorescence signals using a fluorometer. Images were obtained using an inverted Nikon ECLIPSE Ts2 microscope (Nikon, Tokyo, Japan).

### 2.6. Immunoblotting

HT-22 cells were seeded at a density of 2 × 10^5^ cells into a 6-well plate. Cells were treated with 10 mM Glutamate with and without affibodies for 0, 4 and 8 h. Cells were washed with PBS and lysed with lysis buffer containing 1% Triton X-100, phosphatase inhibitor and a protease inhibitor cocktail and inverting for 1 h at 4 °C. The supernatant was collected, and LDS buffer was added for gel electrophoresis. Protein samples were then loaded onto the protein gel and Western blotting was conducted with the following antibodies, TMEM16F (Human protein atlas, Stockholm, Sweden), penta-His tag (Qiagen, Hilden, Germany), ERK (R&D systems, Minneapolis, MN, USA), phosphor-ERK (Cell signaling, Danvers, MA, USA), p38 (Cell signaling), phosphor-p38 (Cell signaling), JNK (Cell signaling), phosphor-JNK (Cell signaling), and actin (Cell signaling).

### 2.7. Scrambling Assay

Liposomes were prepared from a soybean polar lipid extract, and 0.5 mol% NBD-labeled phosphoethanolamine (PE) was added to the lipid mixture. Lipids were dissolved in reconstitution buffer (300 mM KCl, 20 mM HEPES, pH 7.4) in the presence of 35 mM 3-((3-cholamidopropyl) dimethylammonio)-1-propanesulfonate (CHAPS). Then, 40 mg of TMEM16F proteins were added to 8 mg of lipids. Proteoliposomes were formed by removing detergent with Bio-Beads SM-2 Adsorbent (Bio-Rad, Hercules, CA, USA). Phospholipid scrambling activity was measured as described in a previous study [23]. After extruding liposomes through a 400 nm membrane, 20 μL liposomes was added to 1.98 mL of scrambling solution (300 mM KCl, 50 mM HEPES, 0.5 mM Ca(NO_3_)_2_ or 2 mM EGTA, pH 7.4). Fluorescent signals were monitored (excitation, 470 nm; emission, 530 nm) with a spectrofluorometer (HORIBA Scientific, Edison, NJ, USA). Finally, 40 μL of sodium dithionite (40 mM final concentration) was added to bleach the NBD fluorophores.

### 2.8. Ca^2+^ Influx Assay

TMEM16F knockout (KO) 293T cells and human TMEM16F overexpressing 293T cells are used for the Ca^2+^ influx assay. TMEM16F KO cells were generated by using CRISPR/Cas9 gene knockout kit (Origene, Rockville, MD, USA). Cells were seeded at a density of 3 × 10^4^ cells into a 96-well plate. Calcium 6 dye (Molecular Devices) was loaded into cells for 2 h according to the manufacturer’s protocol. Then, to induce the channel activation, 25 mM PFA/2 mM DTT was treated as described in previous report [16]. The fluorescence measurements were performed using Flexstation3 (Molecular Device) at an excitation wavelength of 485 nm and an emission wavelength of 525 nm. As a negative control, fluorescent signal from TMEM16F knockout 293T cells was monitored. To specifically assess TMEM16F-dependent Ca^2+^ influx, the fluorescence signals observed in TMEM16F knockout cells were subtracted.

## 3. Results

### 3.1. Expression of hTMEM16F-Specific Affibodies and Human TMEM16 Proteins

**Figure 1:**
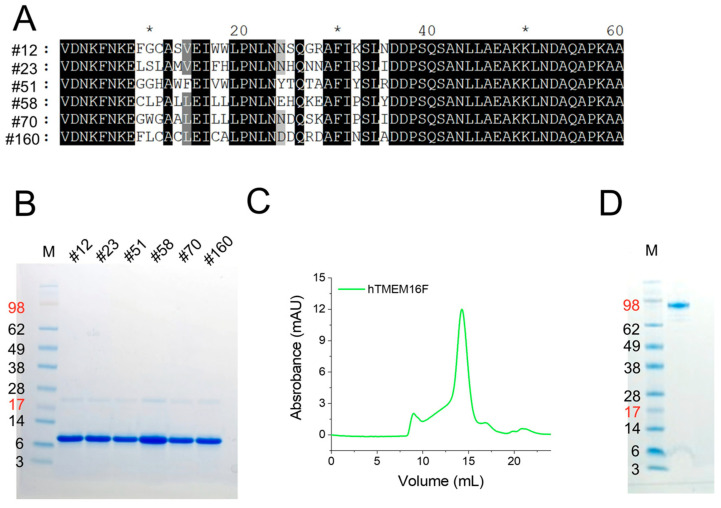
Screening for TMEM16F-specific affibodies using phage display. (**A**) The amino acid sequences of the selected six affibodies. (**B**) Expression of candidate affibodies. The six selected affibodies were purified and their purity was confirmed by SDS-PAGE and Coomassie blue staining. A total of 2 mg of each affibody was loaded into each lane. (**C**) FPLC profile of purified TMEM16F proteins. After purifying human TMEM16F, SEC was performed on a Superose 6 column. (**D**) SDS-PAGE and Coomassie blue staining of hTMEM16F after FPLC.

In our previous study, we isolated novel TMEM16F affibodies by biopanning a phage display library for TMEM16F-binding affibodies, yielding 98 candidate affibodies. Of these, 38 were sequenced and two of these were subjected for further analysis and shown to bind with high affinity to TMEM16F [22]. In present study, we selected six other affibodies from among the 38 previously sequenced affibody candidates, (Figure 1A), and tried to characterize their binding to purified TMEM16F protein. The selected affibody genes were synthesized and cloned into a bacterial expression vector with a C-terminal His tag sequence for purification. Affibody expression was induced by IPTG at 37 °C for 3 h. Six affibodies from 1 L cultures were purified using cobalt column chromatography. After removal of residual imidazole using desalting column, the purified proteins were concentrated using a centrifugal filter unit and subjected to SDS-PAGE. Most of the protein migrated near 6 kDa as expected (Figure 1B). To test the binding of candidate affibodies, human TMEM16F proteins were expressed in 293 cells (GnTI-) and purified by using affinity chromatography. The eluted proteins were separated by size exclusion chromatography (Figure 1C). A monodisperse peak of human TMEM16F protein was collected and subjected to SDS-PAGE (Figure 1D).

### 3.2. Interaction of Candidate Affibodies with hTMEM16F Protein

**Figure 2:**
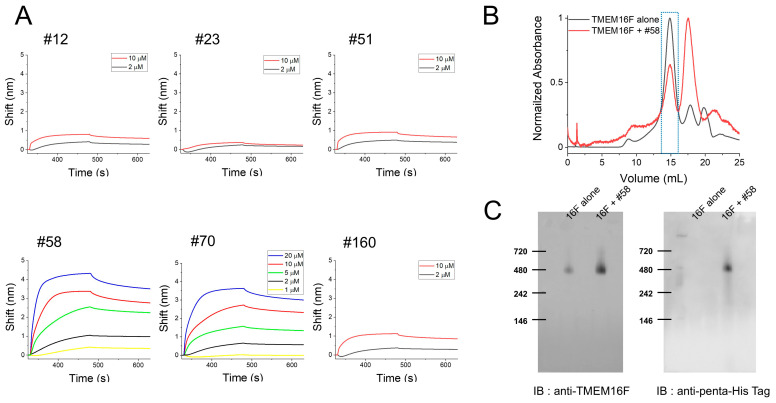
Binding of candidate affibodies to purified human TMEM16F proteins. (**A**) BLI instrument measurements of the binding affinities of the candidate affibodies. TMEM16F protein (3 mM) was immobilized on a streptavidin (SA) sensor and each affibody was injected to test how it interacts with TMEM16F. Among the six affibodies, two affibodies, #58 and #70, showed higher affinity and were injected again at three to four concentrations to estimate their Kd values. (**B**) Superose 6 FPLC elution profiles of a mixture ofTMEM16F and #58 affibody and TMEM16F alone. The affibody and TMEM16F were mixed at 4 °C for 3 h, and then separated by Superose 6 FPLC. (**C**) After FPLC, the presence of TMEM16F and the affibody in the same fraction was confirmed by Blue Native PAGE followed by Western blotting.

The binding of the affibodies to TMEM16F protein was directly monitored using biolayer interferometry (BLI). Since the SBP-tag used for protein purification can bind to streptavidin (SA)-conjugated biosensors, purified hTMEM16F proteins were directly immobilized on the SA biosensor. Initially, we briefly applied two concentrations of the affibodies (2 mM and 10 mM) to monitor their association with and dissociation from TMEM16F, which revealed that two affibodies, #58 and #70, caused higher wavelength shifts than those of the other four affibodies (Figure 2A). Finally, to estimate their binding affinities, various concentrations of these two affibodies (#58 and #70) were injected to monitor their binding to and release from TMEM16F (Figure 2A). #58 and #70 affibodies showed relatively high binding affinity (Kd) for TMEM16F with Kd values of 0.3 and 0.5 mM, respectively. Next, we examined the specificities of the two affibodies (#58 and #70) by investigating whether they bound to the related TMEM16 family members TMEM16A and TMEM16K (Supplement Figure S1). After purification of the human TMEM16A (hTMEM16A) and human TMEM16K (hTMEM16K), each tagged with SBP, the two proteins were directly immobilized on SA biosensors. Injection with 2 mM and 10 mM of these affibodies and measurement of the wavelength shifts showed that unlike their high affinity binding to TMEM16F, two affibodies could not bind well to either TMEM16A or TMEM16K proteins (Supplemental Figure S1).

Next, we investigated whether these affibodies bound to native TMEM16F by performing size exclusive chromatography (SEC) on a mixture of TMEM16F and high affinity affibody (#58) and TMEM16F alone. After incubating purified TMEM16F protein with #58 affibody for 2 h at 4 °C, the mixture was separated by Superose 6 fast protein liquid chromatography (FPLC) (Figure 2B). Blue Native PAGE followed by Western blot analyses of the fractions eluted around 14.9 mL revealed that the #58 affibody co-eluted with TMEM16F near 480 kDa, as reported in previous studies [24,25] (Figure 2C). These results strongly suggest that this affibody also binds to native TMEM16F protein. Together, the results indicate that the candidate affibodies bind to TMEM16F.

### 3.3. Effects of the Candidate TMEM16F Affibodies on Neuronal Cell Death Induced by Glutamate

**Figure 3:**
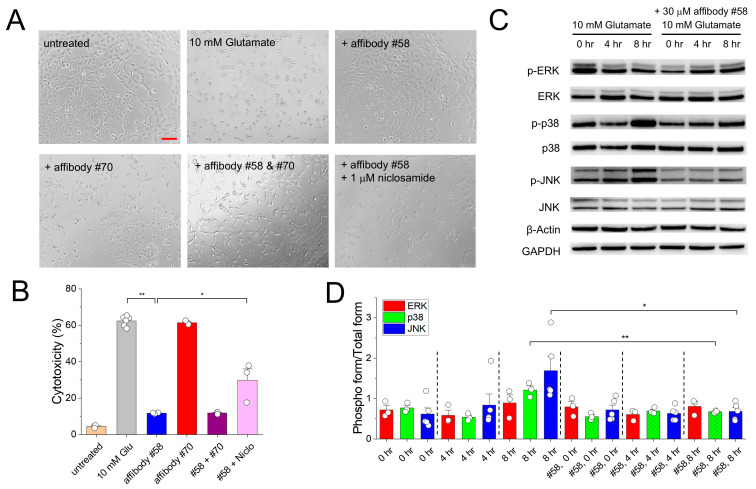
Effect of candidate TMEM16F affibodies on glutamate-induced neuronal cell death. (**A**) Representative images of HT-22 mouse neuronal cells after treatment with 10 mM glutamate with and without 30 mM of the candidate affibodies, #58 and #70. To inhibit the TMEM16F function, 1 mM niclosamide was also treated. Scale bar, 100 mm (**B**) Cytotoxicity was calculated by measuring the amount of lactate dehydrogenase (LDH) release. As negative and positive controls, untreated and 10 mM glutamate-treated HT-22 cells were used. (**C**) Immunoblotting of HT-22 mouse neuronal cell lysates after treatment with 10 mM Glutamate for 0, 4, and 8 h with and without 30 mM of the candidate affibodies, #58 and #70. To test whether the affibodies attenuated the activation of MAPK pathways induced by cell death, the amounts of the phosphor-forms and total forms of ERK, p38 and JNK were measured following immunoblotting of HT-22 cell extracts. (**D**) Quantification of band intensities. One-way ANOVA analysis was performed and *p*-values between control (no affibody) and #58 affibody treatment were calculated by Fisher’s test using Origin 2020 (OriginLab). Error bars indicate SEM. ** *p* < 0.01 and * *p* < 0.05.

To enable the practical application of these affibodies, it is essential not only to confirm their high-affinity binding to TMEM16F, but also to investigate whether they influence TMEM16F function. Thus, after confirming the interactions between the two affibodies and TMEM16F by using BLI and SEC, we determined whether the affibodies could act as modulators of TMEM16F-related processes by examining their effects on glutamate-induced oxidative stress in HT-22 cells, a mouse neuronal cell line. To induce cell death, HT-22 cells were treated with 10 mM glutamate (pH 7.4) for 14 h. Compared with the glutamate-untreated cells, 10 mM glutamate-treated cells showed clear evidence of cell death (Figure 3A), as reported in other studies [26,27]. However, when HT-22 cells were incubated under the same conditions in the presence of affibody #58, no significant cell death was observed. Conversely, affibody #70 did not protect HT-22 cells from the cell damage induced by glutamate. To evaluate the cytotoxicity of the affibodies, we measured LDH release from HT-22 cells. Treatment with 10 mM glutamate alone induced the death of 62% of HT-22 cells whereas treatment with glutamate in the presence of 30 mM of affibody #58 reduced cytotoxicity to 12% (Figure 3B). We also confirmed that co-treatment with the affibody #58 and #70 resulted in a reduction in cell death similar to that observed with affibody #58 alone (Figure 3A,B). To determine whether this protective effect against cell death is a direct consequence of altered TMEM16F function, we examined the impact on cell death after co-treating affibody #58 with niclosamide, a well-known TMEM16F inhibitor. To exclude the possibility that niclosamide itself induces cytotoxicity, we treated HT-22 cells with 1, 5, and 10 µM niclosamide and monitored cell viability. While 1 µM niclosamide did not affect cell death, treatment with 5 and 10 µM resulted in noticeable cytotoxic effects (Supplement Figure S4). Quantitative analysis revealed that high concentrations of niclosamide increased cell death by up to 20% (Supplement Figure S4). Based on these results, we selected 1 µM niclosamide for subsequent experiments. When applied together with glutamate and the affibody #58, 1 µM niclosamide increased cell death by 34% (Figure 3B). These findings indicate that niclosamide inhibits TMEM16F function and thereby reduces the protective effect of the affibody #58 on cell death, providing strong evidence that the affibody #58-mediated protection against cell death is achieved through modulation of TMEM16F activity. Besides these two affibodies, we also investigated whether two previously reported affibodies, #50 and #119, with higher binding affinities for TMEM16F than those of #58 and #70, would reduce cell death induced by glutamate. However, neither of these two affibodies was effective in preventing neuronal cell death (Supplement Figure S2A,B).

Excessive glutamate, an excitatory neurotransmitter, can trigger the production of reactive oxygen species (ROS) in neuronal cells, leading to potential damage and neurodegeneration [28]. Since ROS activates Mitogen-Activated Protein Kinase (MAPK) pathways to induce cell death [29], we investigated whether the TMEM16F affibodies would attenuate ERK, p38, and JNK signaling activation induced by glutamate-induced cell death. HT-22 cells were treated with 10 mM glutamate with and without the TMEM16F affibodies for 0, 4 and 8 h, harvested, and subjected to immunoblotting. Among the three MAPKs, p38 and JNK signaling was increased significantly by glutamate treatment, (*p*-value < 0.01), as evidenced by the increases in the phosphor-forms of p38 and JNK with glutamate treatment time, but co-treatment with 30 mM of affibody #58 reduced the levels of the phosphor-forms of p38 and JNK, indicating that it was effective in attenuating the glutamate-induced activation of p38 and JNK signaling (Figure 3C). Based on the band intensities, the #58 affibody reduced by 2-fold (*p*-value < 0.01) and 2.5-fold (*p*-value < 0.05) the levels of the phosphor-forms of p38 and JNK, respectively, relative to those in the presence of glutamate alone (Figure 3D). Together, these results indicate that treatment with affibody #58 can protect neuronal cells from cell death induced by glutamate through MAPK signaling blockade.

### 3.4. Effects of Candidate TMEM16F Affibodies on Microglial Phagocytosis

**Figure 4:**
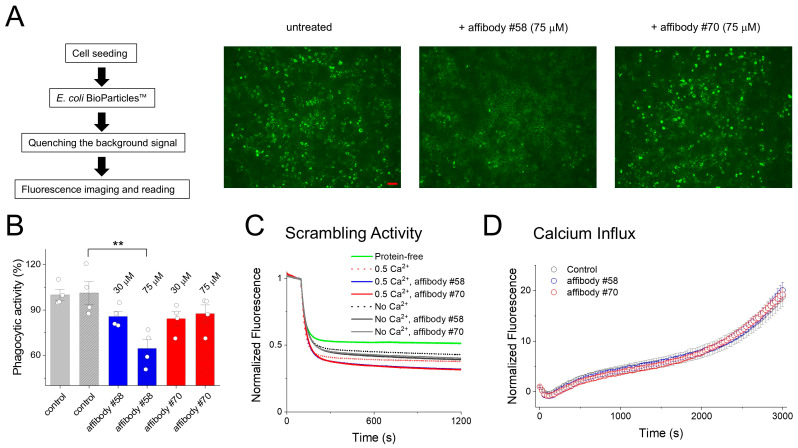
Effect of candidate TMEM16F affibodies on phagocytic activity of microglial cells. (**A**) Representative images of BV2 cells after their exposure to *E. coli* Bioparticles^TM^ in the absence or presence of TMEM16F affibodies. To test the effect of the affibodies on the phagocytic activity of microglial cells, 30 mM and 75 mM of affibodies (#58 and #70) were applied to the cells and phagocytic activity was monitored using fluorescent microscopy. Scale bar, 100 mm. (**B**) Quantification of phagocytic activity of BV2 cells based on the intensity of the fluorescent signals measured using Flexstation3. (**C**) Effects of 20 mM each of #58 and #70 affibodies on TMEM16F scrambling activity in liposomes. Scrambling activity was measured in the absence or presence of 0.5 mM Ca^2+^. (**D**) Effects of 20 mM each of #58 and #70 affibodies on TMEM16F channel activity in 293T cells. Fluorescence from Ca^2+^ dye (Calcium 6) was monitored by using Flexstation 3. To monitor TMEM16F-dependent Ca^2+^ influx, fluorescence signals observed in TMEM16F knockout cells were subtracted. Error bars indicate SEM. ** *p* < 0.01.

A previous report suggested that TMEM16F is an essential component of the microglial response to injury and is important for microglial phagocytosis [30]. Thus, we tested whether TMEM16F affibodies would influence the phagocytic activity of microglial cells by exposing mouse microglial cells (BV2) to *E. coli* Bioparticles^TM^ with and without 30 and 75 μM of affibodies for 1 h. Then, we monitored the engulfment of the *E. coli* Bioparticles^TM^, a measure of microglial phagocytosis. Fluorescent images showed that *E. coli* Bioparticles^TM^ were effectively engulfed by BV2 cells (Figure 4A), but that the fluorescence was reduced when the cells were exposed concomitantly to 75 mM of affibody #58 (Figure 4A). Fluorometry of the fluorescence signals of *E. coli* Bioparticles^TM^ showed that phagocytic activities following treatment with 30 mM and 75 mM of affibody #58 were reduced to 86% and 65%, respectively (Figure 4B), but only the effect of 75 mM of the #58 affibody was statistically significant (*p*-value < 0.01). In the case of affibody #70, slight decreases in phagocytic activity were observed at 30 and 75 mM, but these decreases were not statistically significant. Besides these two affibodies, we tested the effects of two previously reported affibodies with higher binding affinities for TMEM16F [22], affibody #50 and #119, on the phagocytic activity of microglial cells. However, these affibodies did not affect the phagocytic activity of these cells (Supplement Figure S2C,D). These results demonstrate that TMEM16F affibodies can inhibit phagocytosis by BV2 cells.

Finally, we investigated the effects of the two affibodies on scrambling activity using proteoliposomes and channel activity using cell-based assay. Unlike in our previous study, the application of 20 mM of affibody #58 or #70 to both sides of the liposomes in the scrambling assays resulted in only a slight increase in scrambling activity in the presence and absence of Ca^2+^ (Figure 4C). Furthermore, Ca^2+^ imaging was employed to assess the ion channel function of TMEM16F. As a negative control, fluorescent signal from TMEM16F knockout 293T cells was monitored by using fluorometer (Supplement Figure S5). Unlike the activation of lipid scrambling by affibody #58 and #70, Ca^2+^ influx through TMEM16F was not influenced by these affibodies (Figure 4D and Supplement Figure S5).

## 4. Discussion

In this study, we selected six other affibodies from among the 38 previously sequenced affibody candidates and subsequently validated the functional effects of two of these affibodies, #58 and #70, on microglia and neuronal cell lines. Unlike traditional high-throughput screening approaches that select compounds based on their functional effects, the identification of affibodies specific to membrane proteins does not inherently mean that they will affect the activity of the targeted membrane protein. Therefore, it is essential to perform additional experiments to determine whether the selected affibodies influence the function of the target protein.

To this end, we examined the effects of the two affibodies not only in proteoliposomes but also at the cellular level by applying them to microglial and neuronal cell lines. Since TMEM16F is implicated in several pathways leading to neuronal damage or death, particularly under pathological conditions such as ischemia and inflammation [31,32], our initial focus was to examine the effect of the affibodies on the regulation of neuronal cell death. Interestingly, we found that treatment with one of the identified affibodies, #58, significantly reduced glutamate-induced cell death of HT-22 cells by blocking MAPK signaling pathway. This result appears to contradict previous reports that TMEM16F expression is upregulated under pathological conditions such as ischemia, where it contributes to neuronal cell death [31]. Furthermore, another study showed that deletion of the TMEM16F gene alleviates neuronal cell death and is beneficial in tauopathy models [32]. However, TMEM16F activation does not necessarily increase cell death. For instance, Ba/F3 cell lines harboring mutations (D430G) that result in constitutive TMEM16F activity do not show any abnormalities in cell proliferation or viability. Furthermore, these cells are not engulfed by macrophages [33]. In addition, in thymocytes treated with LLO (listeriolysin O), cell death was observed only in TMEM16F-knockout cell lines, while wild-type cells remained viable [10]. This suggests that TMEM16F activity can mediate membrane repair, protecting cells by facilitating the release of extracellular vesicles containing damaged membranes. Thus, to gain a better understanding of our results, it appears important to identify a potential link between TMEM16F activation and the MAPK signaling pathway for future research.

Taken together, these apparently contradictory results highlight the complex roles of TMEM16F in different cellular contexts. Nevertheless, previous findings have consistently shown that TMEM16F function is essential in neuronal cell death, as evidenced by siRNA-mediated knockdown or genetic knockout approaches, which significantly attenuate neuronal death [31,32]. However, these approaches primarily operate through changes in TMEM16F expression levels, leaving unresolved the critical question of whether direct functional modulation of TMEM16F is sufficient to alter neuronal survival outcomes. To date, no selective modulators of TMEM16F have been reported, and no pharmacological activators are available. The absence of such tools has made it difficult to directly test how functional regulation of TMEM16F contributes to neuronal pathology. In this regard, our study provides novel evidence that TMEM16F-specific affibodies can modulate neuronal survival through functional regulation of TMEM16F activity, thereby offering a new way to study TMEM16F proteins and to consider possible therapeutic approaches that target TMEM16F.

According to our findings, treatment with one of the affibodies, #58, predicted to increase TMEM16F function in the mouse microglial cell line, BV2, significantly decreased engulfment of bioparticles by these cells. This observation also appears to contradict previous results, in which knockout of TMEM16F in mouse spinal microglia led to morphological changes and impaired phagocytic activity [30,34]. However, our results represent the first direct evidence of the effects of a candidate TMEM16F activator on microglial cell lines, unlike in previous studies where the effects were measured using a TMEM16F knockout model. Moreover, a recent study using BV2 cells and primary microglial cells showed that knockout of TMEM16F leads to increased phagocytic activity [35]. These findings are consistent with our studies and may provide further insight into how modulation of TMEM16F affects the phagocytic activity of microglial cells.

An interesting observation is that while both #58 and #70 affibodies had similar effects in liposome-based assays, only the #58 affibody had a significant effect on microglial and neuronal cells. This difference between the two affibodies may be due to potential differences in the binding sites of the two affibodies, with one potentially interacting with the extracellular domain and the other with the intracellular domain of the TMEM16F protein. To validate this hypothesis, it will be important to determine the three-dimensional structures of the affibodies in complex with TMEM16F, which could provide a structural explanation for the differences in their cellular activities. The complex structure of TMEM16F and affibody will also be necessary to obtain critical insights into the binding sites of the affibodies for future drug targeting of TMEM16F. This information would help uncover the molecular mechanisms of action of these potential modulators, which could then serve as a foundation for the discovery and development of novel TMEM16F regulators. If the binding site of the affibodies is located on the intracellular side of the PM, this could limit its accessibility and functional efficacy in live-cell application. To address potential issues in the cell permeability of the affibodies, they could be either conjugated to cell-penetrating peptide (CPP) or expressed intracellularly. These strategies would enhance their ability to access intracellular targets and facilitate their functional validation in a physiologically relevant context. Ultimately, this approach may broaden the applicability of affibody-based therapeutics and research tools for intracellular modulation of TMEM16F.

In summary, we identified additional TMEM16F-specific affibodies that bind specifically to TMEM16F with high affinity. One of these affibodies could modulate neuronal cell death and phagocytic activity of microglial cells. This is the first study to demonstrate that TMEM16F-specific affibodies could function as modulators of TMEM16F activation in cellular level. Further characterization of how the affibodies affect TMEM16F function will be required before they can be used for therapeutic purposes.

## Data Availability

The original contributions presented in this study are included in the article and supplementary material. Further inquiries can be directed to the corresponding author.

## Supplementary Materials

The following supporting information can be downloaded at: https://www.mdpi.com/article/10.3390/membranes15090255/s1, Figure S1: The purification of TMEM16 proteins and the specificity of TMEM16F affibodies on TMEM16 proteins; Figure S2: Effects of candidate affibodies on neuronal and microglial cells; Figure S3: Whole Western blots; Figure S4. Effects of niclosamide on HT-22 cells; Figure S5. Effects of candidate affibodies on channel activity of TMEM16F.

## References

[1] Pomorski T., Menon A.K. (2006). Lipid flippases and their biological functions. Cell Mol. Life Sci..

[2] Bevers E.M., Williamson P.L. (2010). Phospholipid scramblase: An update. FEBS Lett..

[3] Balasubramanian K., Schroit A.J. (2003). Aminophospholipid asymmetry: A matter of life and death. Annu. Rev. Physiol..

[4] Suzuki J., Fujii T., Imao T., Ishihara K., Kuba H., Nagata S. (2013). Calcium-dependent phospholipid scramblase activity of TMEM16 protein family members. J. Biol. Chem..

[5] Suzuki J., Imanishi E., Nagata S. (2014). Exposure of phosphatidylserine by Xk-related protein family members during apoptosis. J. Biol. Chem..

[6] Ernst O.P., Menon A.K. (2015). Phospholipid scrambling by rhodopsin. Photochem. Photobiol. Sci..

[7] Jan L.Y., Jan Y.N. (2025). Wide-ranging cellular functions of ion channels and lipid scramblases in the structurally related TMC, TMEM16 and TMEM63 families. Nat. Struct. Mol. Biol..

[8] Yang H., Kim A., David T., Palmer D., Jin T., Tien J., Huang F., Cheng T., Coughlin S.R., Jan Y.N. (2012). TMEM16F forms a Ca2+-activated cation channel required for lipid scrambling in platelets during blood coagulation. Cell.

[9] Hu Y., Kim J.H., He K., Wan Q., Kim J., Flach M., Kirchhausen T., Vortkamp A., Winau F. (2016). Scramblase TMEM16F terminates T cell receptor signaling to restrict T cell exhaustion. J. Exp. Med..

[10] Wu N., Cernysiov V., Davidson D., Song H., Tang J., Luo S., Lu Y., Qian J., Gyurova I.E., Waggoner S.N. (2020). Critical Role of Lipid Scramblase TMEM16F in Phosphatidylserine Exposure and Repair of Plasma Membrane after Pore Formation. Cell Rep..

[11] Castoldi E., Collins P.W., Williamson P.L., Bevers E.M. (2011). Compound heterozygosity for 2 novel TMEM16F mutations in a patient with Scott syndrome. Blood.

[12] Ousingsawat J., Wanitchakool P., Schreiber R., Kunzelmann K. (2018). Contribution of TMEM16F to pyroptotic cell death. Cell Death Dis..

[13] Cui Z.Q., Hu X.Y., Yang T., Guan J.W., Gu Y., Li H.Y., Zhang H.Y., Xiao Q.H., Sun X.H. (2023). TMEM16F may be a new therapeutic target for Alzheimer’s disease. Neural Regen. Res..

[14] Braga L., Ali H., Secco I., Chiavacci E., Neves G., Goldhill D., Penn R., Jimenez-Guardeno J.M., Ortega-Prieto A.M., Bussani R. (2021). Drugs that inhibit TMEM16 proteins block SARS-CoV-2 spike-induced syncytia. Nature.

[15] Lam A.K.M., Rutz S., Dutzler R. (2022). Inhibition mechanism of the chloride channel TMEM16A by the pore blocker 1PBC. Nat. Commun..

[16] Feng S., Puchades C., Ko J., Wu H., Chen Y., Figueroa E.E., Gu S., Han T.W., Ho B., Cheng T. (2023). Identification of a drug binding pocket in TMEM16F calcium-activated ion channel and lipid scramblase. Nat. Commun..

[17] Cabrita I., Benedetto R., Schreiber R., Kunzelmann K. (2019). Niclosamide repurposed for the treatment of inflammatory airway disease. J. Clin. Investig..

[18] Centeio R., Cabrita I., Benedetto R., Talbi K., Ousingsawat J., Schreiber R., Sullivan J.K., Kunzelmann K. (2020). Pharmacological Inhibition and Activation of the Ca^2+^ Activated Cl^−^ Channel TMEM16A. Int. J. Mol. Sci..

[19] Miner K., Labitzke K., Liu B., Wang P., Henckels K., Gaida K., Elliott R., Chen J.J., Liu L., Leith A. (2019). Drug Repurposing: The Anthelmintics Niclosamide and Nitazoxanide Are Potent TMEM16A Antagonists That Fully Bronchodilate Airways. Front. Pharmacol..

[20] Liang P., Wan Y.C.S., Yu K., Hartzell H.C., Yang H. (2024). Niclosamide potentiates TMEM16A and induces vasoconstriction. J. Gen. Physiol..

[21] Sim J.R., Shin D.H., Park P.G., Park S.H., Bae J.Y., Lee Y., Kang D.Y., Kim Y.J., Aum S., Noh S.H. (2022). Amelioration of SARS-CoV-2 infection by ANO6 phospholipid scramblase inhibition. Cell Rep..

[22] Kim E., Bang J., Sung J.H., Lee J., Shin D.H., Kim S., Lee B.C. (2023). Generation of human TMEM16F-specific affibodies using purified TMEM16F. Front. Mol. Biosci..

[23] Lee B.C., Menon A.K., Accardi A. (2016). The nhTMEM16 Scramblase Is Also a Nonselective Ion Channel. Biophys. J..

[24] Ishihara K., Suzuki J., Nagata S. (2016). Role of Ca(2+) in the Stability and Function of TMEM16F and 16K. Biochemistry.

[25] Watanabe R., Sakuragi T., Noji H., Nagata S. (2018). Single-molecule analysis of phospholipid scrambling by TMEM16F. Proc. Natl. Acad. Sci. USA.

[26] Akanda M.R., Kim M.J., Kim I.S., Ahn D., Tae H.J., Rahman M.M., Park Y.G., Seol J.W., Nam H.H., Choo B.K. (2018). Neuroprotective Effects of Sigesbeckia pubescens Extract on Glutamate-Induced Oxidative Stress in HT22 Cells via Downregulation of MAPK/caspase-3 Pathways. Cell Mol. Neurobiol..

[27] Zhang W., Sun H., Zhao W., Li J., Meng H. (2024). Suppression of JNK pathway protects neurons from oxidative injury via attenuating parthanatos in glutamate-treated HT22 neurons. Sci. Rep..

[28] Savolainen K.M., Loikkanen J., Eerikainen S., Naarala J. (1998). Interactions of excitatory neurotransmitters and xenobiotics in excitotoxicity and oxidative stress: Glutamate and lead. Toxicol. Lett..

[29] Son Y., Cheong Y.K., Kim N.H., Chung H.T., Kang D.G., Pae H.O. (2011). Mitogen-Activated Protein Kinases and Reactive Oxygen Species: How Can ROS Activate MAPK Pathways?. J. Signal Transduct..

[30] Batti L., Sundukova M., Murana E., Pimpinella S., De Castro Reis F., Pagani F., Wang H., Pellegrino E., Perlas E., Di Angelantonio S. (2016). TMEM16F Regulates Spinal Microglial Function in Neuropathic Pain States. Cell Rep..

[31] Zhang Y., Li H., Li X., Wu J., Xue T., Wu J., Shen H., Li X., Shen M., Chen G. (2020). TMEM16F Aggravates Neuronal Loss by Mediating Microglial Phagocytosis of Neurons in a Rat Experimental Cerebral Ischemia and Reperfusion Model. Front. Immunol..

[32] Zubia M.V., Yong A.J.H., Holtz K.M., Huang E.J., Jan Y.N., Jan L.Y. (2024). TMEM16F exacerbates tau pathology and mediates phosphatidylserine exposure in phospho-tau-burdened neurons. Proc. Natl. Acad. Sci. USA.

[33] Segawa K., Suzuki J., Nagata S. (2011). Constitutive exposure of phosphatidylserine on viable cells. Proc. Natl. Acad. Sci. USA.

[34] Zhao J., Gao Q.Y. (2019). TMEM16F inhibition limits pain-associated behavior and improves motor function by promoting microglia M2 polarization in mice. Biochem. Biophys. Res. Commun..

[35] Zubia M.V. (2021). Multifaceted Roles of the Lipid Scramblase TMEM16F in Tauopathy. Ph.D. Thesis.

