# Supplementary material for: Characterization of TMEM16F-Specific Affibodies and Their Cellular Effects
Source: Membranes (Basel). 2025 Aug 28;15(9):255. doi: 10.3390/membranes15090255 (PMC12471793; doi:10.3390/membranes15090255)
Supplement: Supplementary file 1 [file membranes-15-00255-s001.zip › membranes-3807655-supplementary.docx]

**
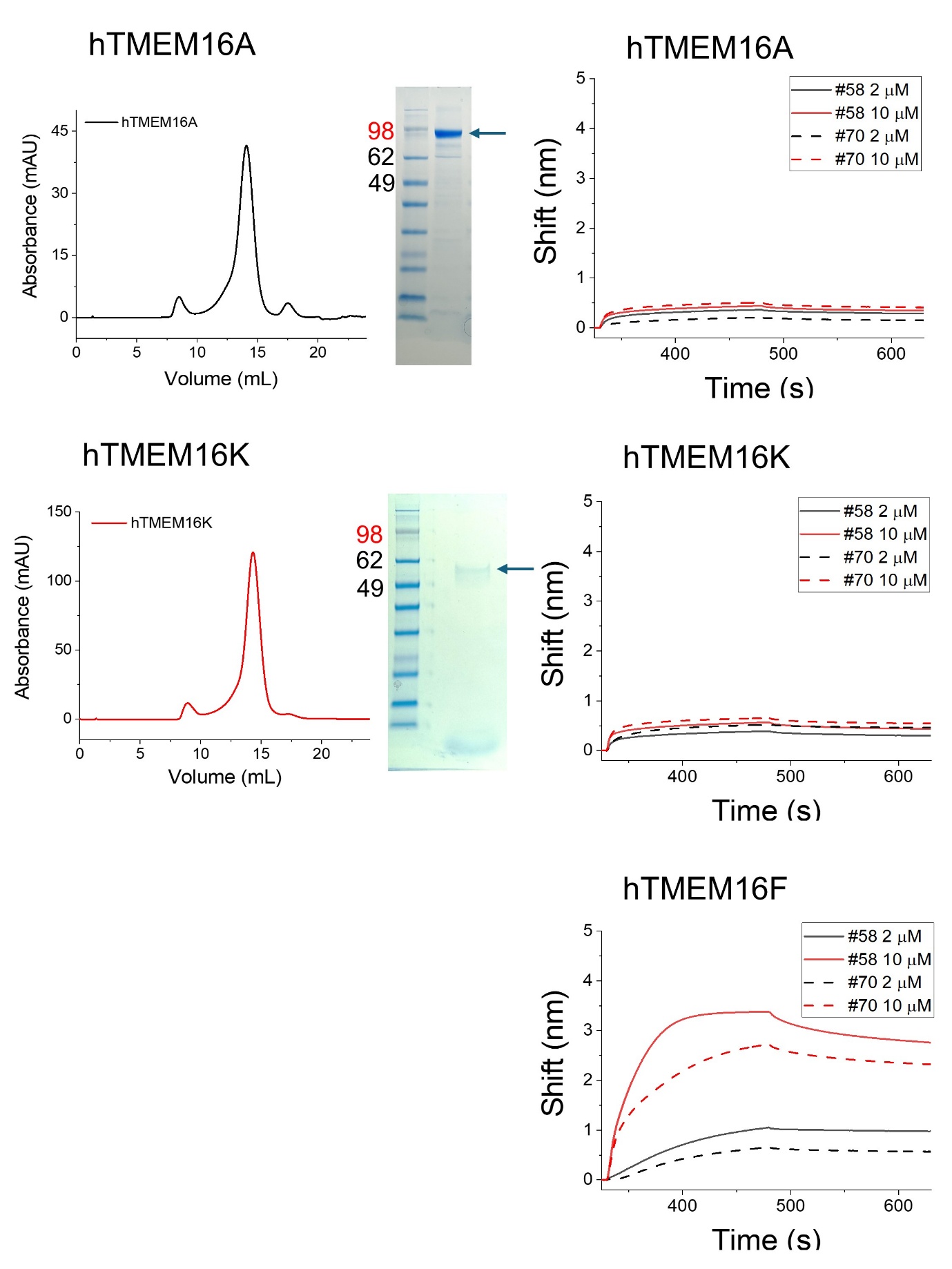
**

**Supplement Figure 1. The purification of TMEM16 proteins and the specificity of TMEM16F affibodies on TMEM16 proteins**

To test the specificity of #58 and #70 affibodies on TMEM16F, closely related TMEM16 family member, TMEM16A and TMEM16K protein were expressed and purified. After immobilizing the TMEM16A and TMEM16K protein into the BLI sensor, binding of candidate affibodies to the TMEM16A was monitored.

**
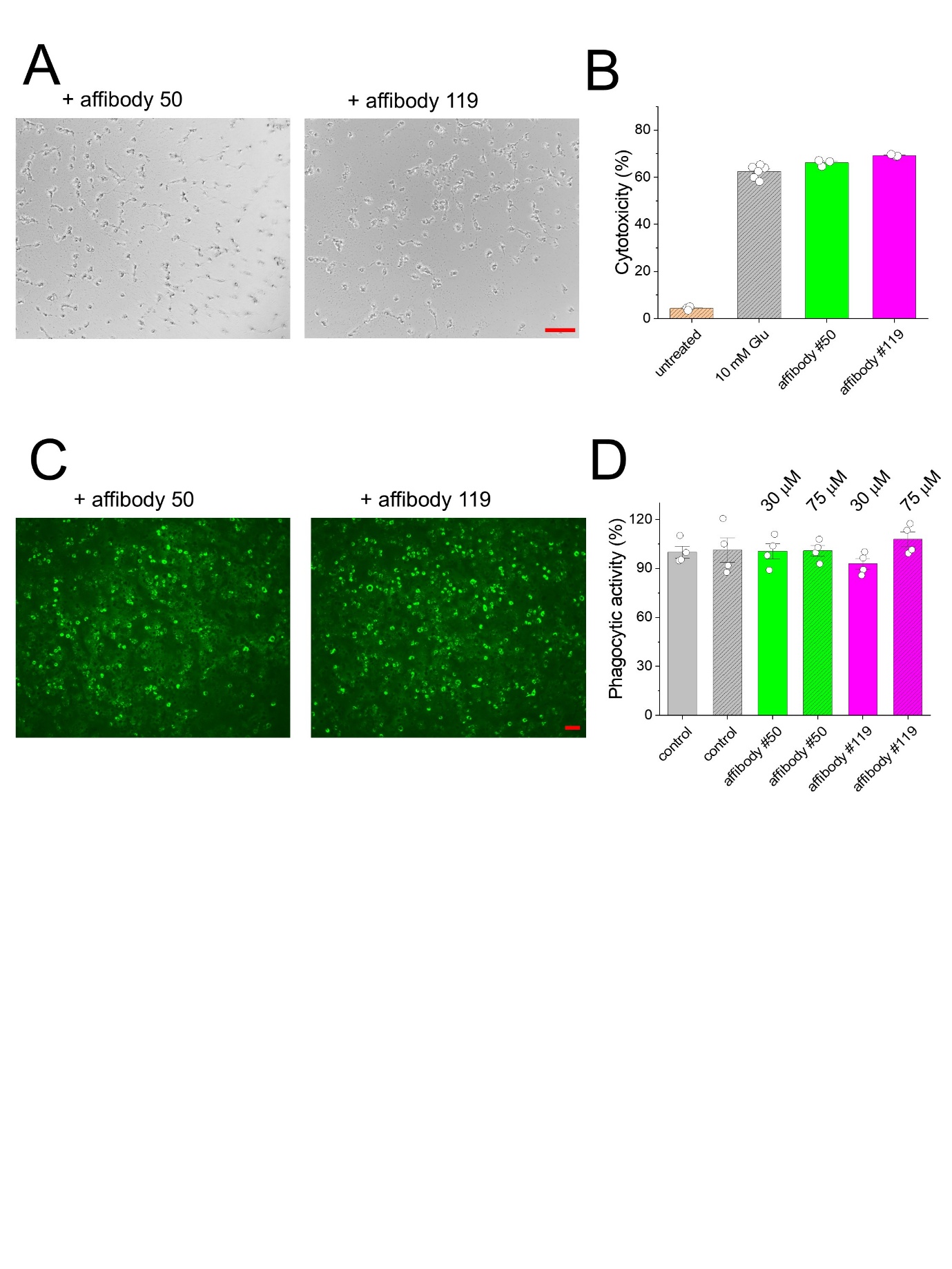
**

**Supplement Figure 2. Effects of candidate affibodies on neuronal and microglial cells**

(A) Representative image of HT-22 mouse neuronal cells after treating 10 mM Glutamate in the presence of 30 μM candidate affibodies, #50 and #119. (B) Cytotoxicity was calculated by measuring the amount of lactate dehydrogenase (LDH) from each condition. As negative and positive controls, untreated and 10 mM glutamate-treated HT-22 cells were used. (C) Representative image of BV2 cells after treating *E.coli* Bioparticles^TM^ in the presence of TMEM16F affibodies. In order to test the effect of affibodies on the phagocytic activity of microglial cells, 30 μM and 75 μM of affibodies (#50 and #119) were treated and phagocytic activity was monitored by using fluorescent microscopy. (D) Quantification of phagocytic activity of BV2 cells by measuring fluorescent signal using Flexstation3.


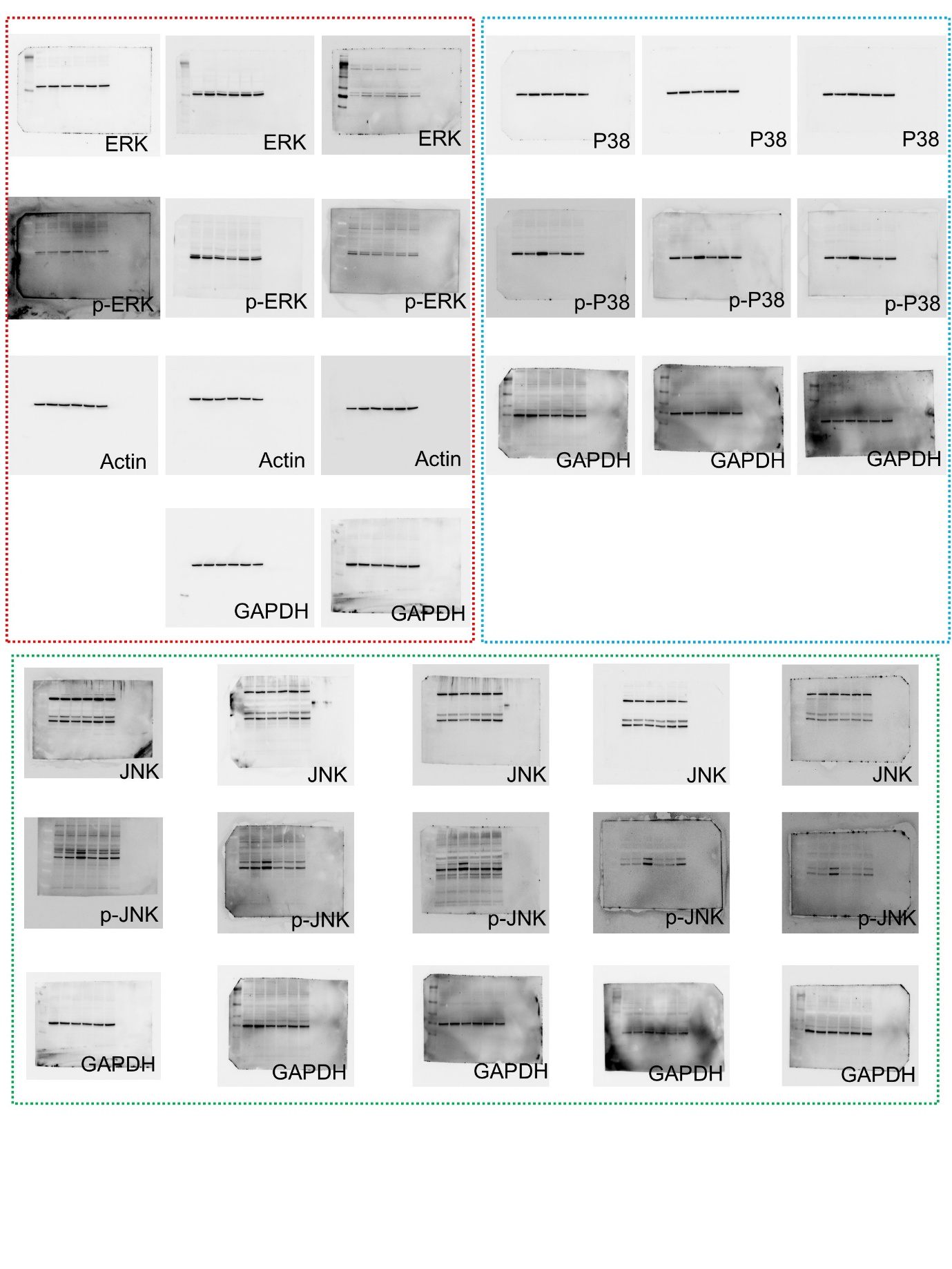
 **Supplement Figure 3. Whole Western Blots**


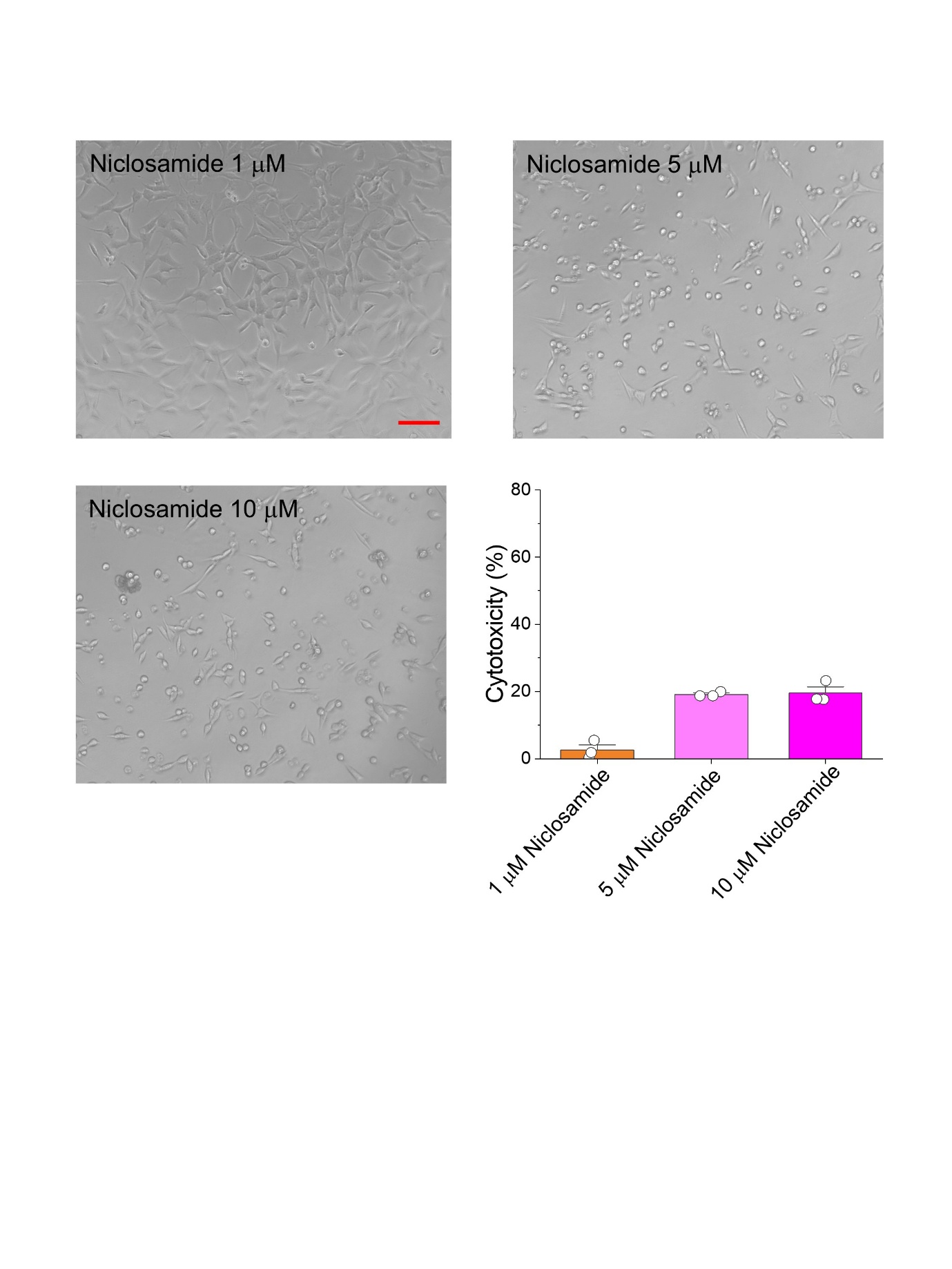


**Supplement Figure 4. Effects of niclosamide on HT-22 cells**

Representative images of HT-22 cells after treatment with 1, 5, 10 μM niclosamide. Cytotoxicity was calculated by measuring the amount of lactate dehydrogenase (LDH) release.


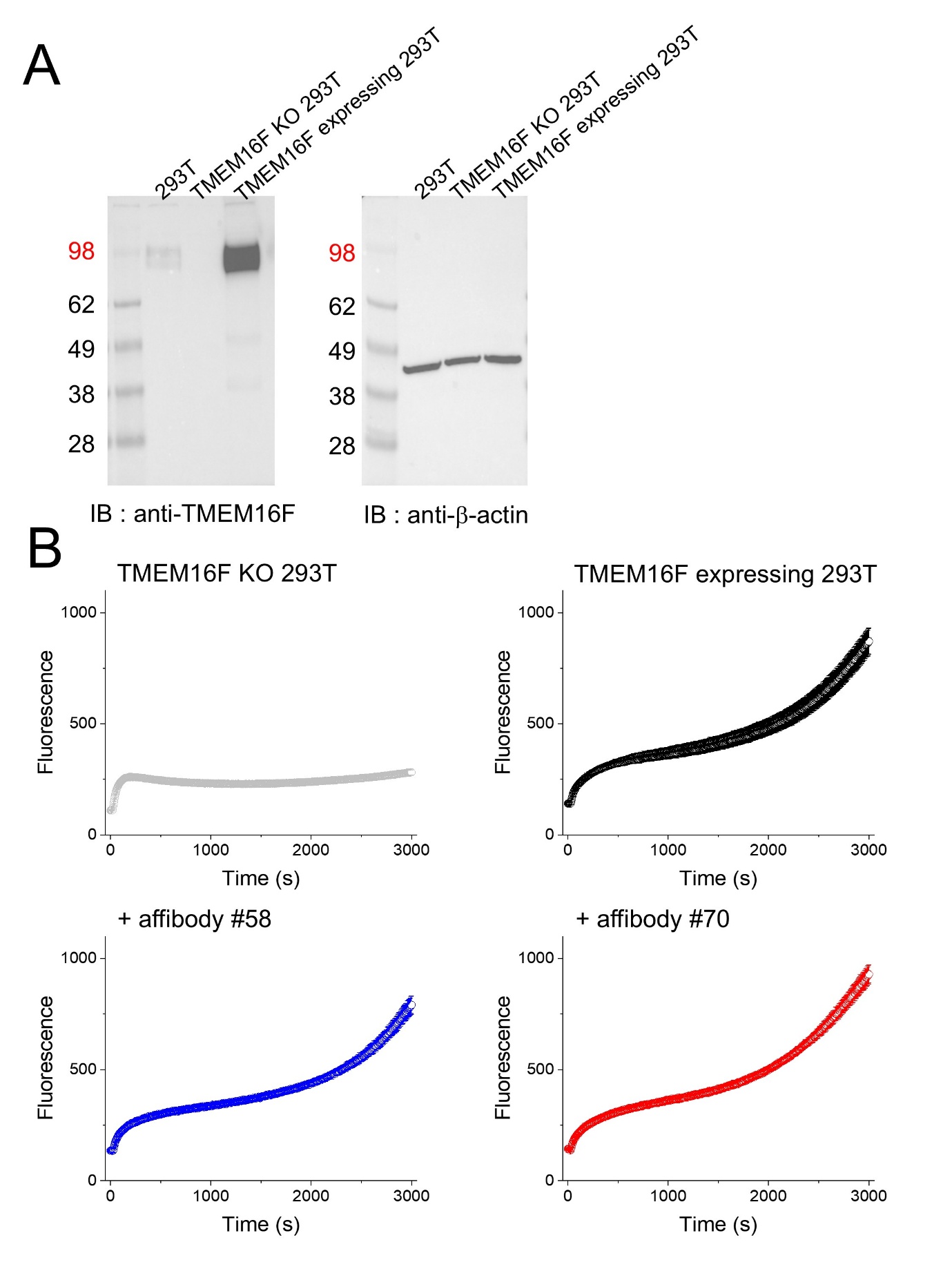


**Supplement Figure 5. Effects of candidate affibodies on channel activity of TMEM16F**

(A) Immunoblotting of 293T cells, TMEM16F knockout (KO) 293T cells and TMEM16F expressing 293T cells. TMEM16F and β-actin antibodies were used to validate the expression of TMEM16F protein. (B) Representative curves of Ca^2+^ imaging for monitoring of TMEM16F channel activity. After loading Ca^2+^ dye (Calcium 6) into 293T cells, PFA and DTT were treated to induce the Ca^2+^ release from intracellular stores. As a negative control, TMEM16F knockout 293T cells were used. The effects of affibody #58 and #70 on channel activity of TMEM16F were monitored by measuring the fluorescence changes using Flexstation 3.
